# Ocular muscle metastasis as the initial presentation of a malignant pheochromocytoma: A unique case

**DOI:** 10.1002/ccr3.2990

**Published:** 2020-05-28

**Authors:** Gholamreza Khataminia, Abdolhasan Talaiezadeh, Ali Bagheri, Pedram Nazari, Amir Mohammad Papan, Nematollah Jazayeri, Roya Baghlani

**Affiliations:** ^1^ Department of Ophthalmology Infectious Ophthalmologic Research Center Ahvaz Jundishapur University of Medical Sciences Ahvaz Iran; ^2^ Department of Surgical Oncology Cancer Research Center Ahvaz Jundishapur University of Medical Sciences Ahvaz Iran; ^3^ Department of Radiation Oncology Interventional Radiotherapy Ward of Golestan Hospital Ahvaz Jundishapur University of Medical Sciences Ahvaz Iran; ^4^ Cancer Research Center Ahvaz Jundishapur University of Medical Sciences Ahvaz Iran; ^5^ Department of Pathology Ahvaz Jundishapur University of Medical Sciences Ahvaz Iran

**Keywords:** malignant, metastasis, ocular muscle, pheochromocytoma

## Abstract

In this paper, we discuss a unique manifestation of malignant pheochromocytoma, which presented with ocular pain. The histopathological study pointed to a possible pheochromocytoma origin. Subsequently, the patient underwent thorough imaging and paraclinical evaluations, which confirmed the diagnosis of pheochromocytoma.

## Graphical abstract

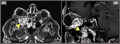

## 1 INTRODUCTION

Malignant pheochromocytoma is a rare malignancy defined as tumor cells at unusual sites, which usually do not harbor chromaffin cells. In this article, we discuss a unique manifestation of malignant pheochromocytoma, which presented with ocular pain as the patient's early complaint and without classic symptoms such as hypertension.

Pheochromocytoma is a type of catecholamine‐releasing adrenal tumor that develops from chromaffin cells.^1^, ^2^ Malignant pheochromocytoma is defined as tumor cells at unusual sites, which usually do not harbor chromaffin cells.^2^ The incidence rate of malignant pheochromocytoma is variable in different reports ranging from 8% to 12.5%.^3^ The diagnosis of metastatic pheochromocytoma is difficult due to lack of definitive histological criteria.^4^

## 2 CASE PRESENTATION

A 47‐year‐old male patient presented to our ophthalmology clinic (Ophthalmology Clinic, Imam Khomeini Hospital) with progressive right eye pain since 2 months ago and painful eye movements, especially on upward gaze. The patient had never experienced paroxysms of headache, palpitation, excessive sweating, flushing, or other commonly associated symptoms of pheochromocytoma. His blood pressure was 125/85 mm Hg on admission. The family history was negative for neuroendocrine diseases, multiple endocrine neoplasia types 1 and 2, and other heredity and possible syndrome‐related tumors. His ophthalmological examinations were normal, except unilateral 23‐mm proptosis.

**Figure 1:**
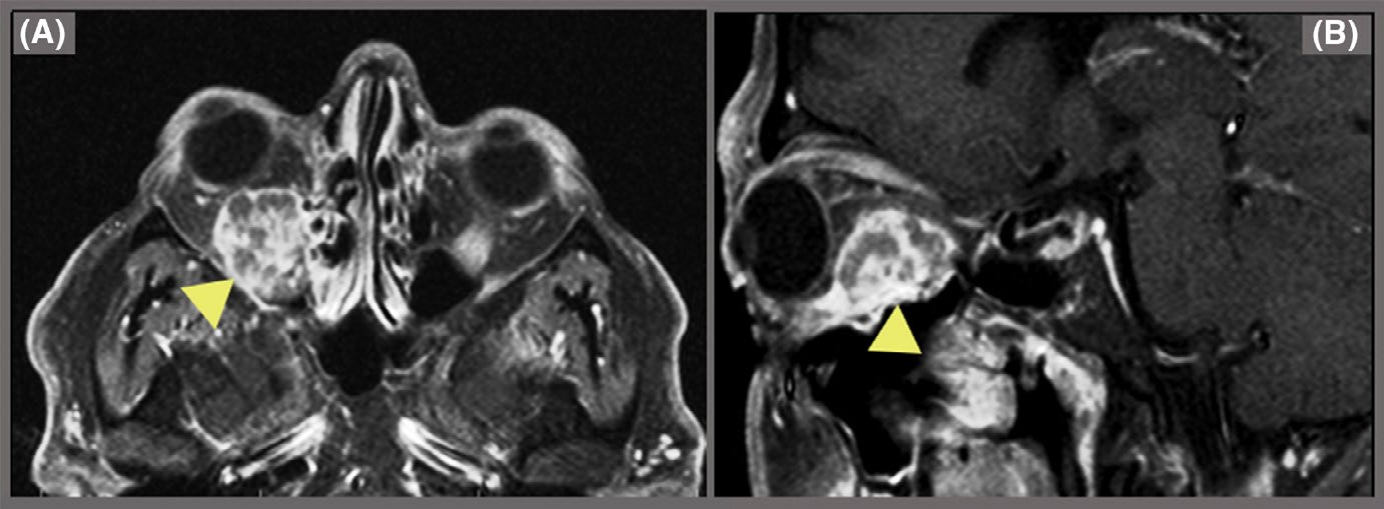
T1‐weighted MRI images of the right orbit show an intracanal space‐occupying lesion, which has caused significant proptosis (A: horizontal view, B: sagittal view)

The patient did not have abnormal hormonal and biochemical values including TSH, T_3_, T_4,_ prolactin, FSH, LH, blood sugar, Na, K, Ca, and phosphor. Thus, we requested an orbital magnetic resonance imaging (MRI; Figure 1). The MRI revealed a 19 × 17 mm intracanal space‐occupying lesion in the inferomedial aspect of the right orbit with mass effect on the optic nerve and upward impression of the right inferior rectus muscle. The mass was biopsied, and subsequent histopathology and immunohistochemistry (ICH) studies were as follows: Predominantly inflamed fibromuscular tissue fragments showed aggregates of polygonal cells with areas of polymorphism and atypical nuclei and eosinophilic cytoplasm, which in turn showed positive immunostaining for synaptophysin (SYN). These findings were in favor of neuroendocrine tumor such as metastasis of malignant pheochromocytoma. Also, the 24‐hours urine study showed a small increase in vanillylmandelic acid and normetanephrine of 8.1 mg/24 h (normal 2‐7 mg/24 h) and 290.1 mcg/24 h (normal: 44‐261 mcg/24 h in normotensive males), respectively.

**Figure 2:**
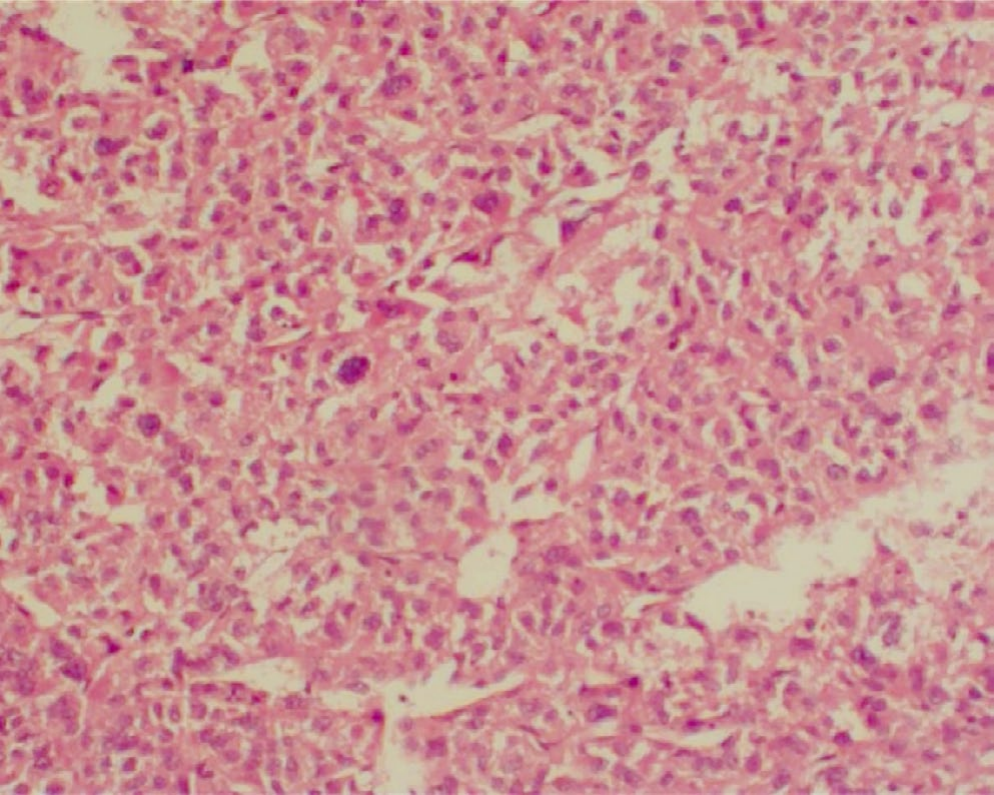
Histopathological examination view of the abdominal pheochromocytoma (H&E stain, magnification ×40)

**Figure 3:**
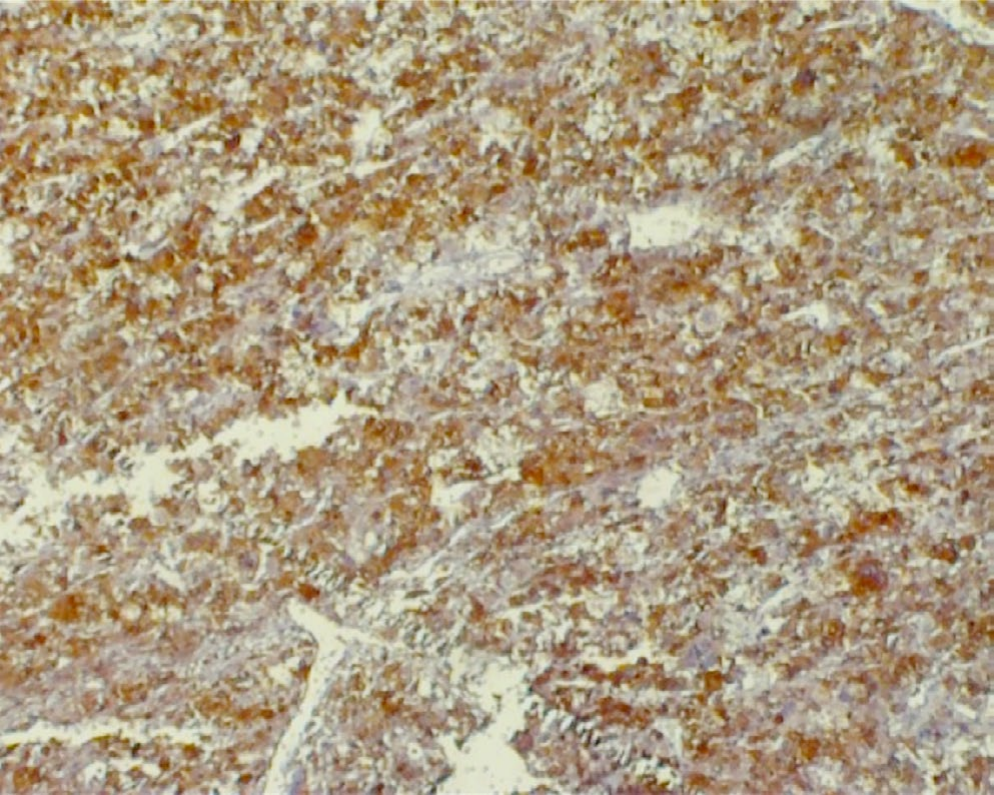
Immunohistochemical study of the adrenal mass specimen: Positive synaptophysin stain is observed

**Figure 4:**
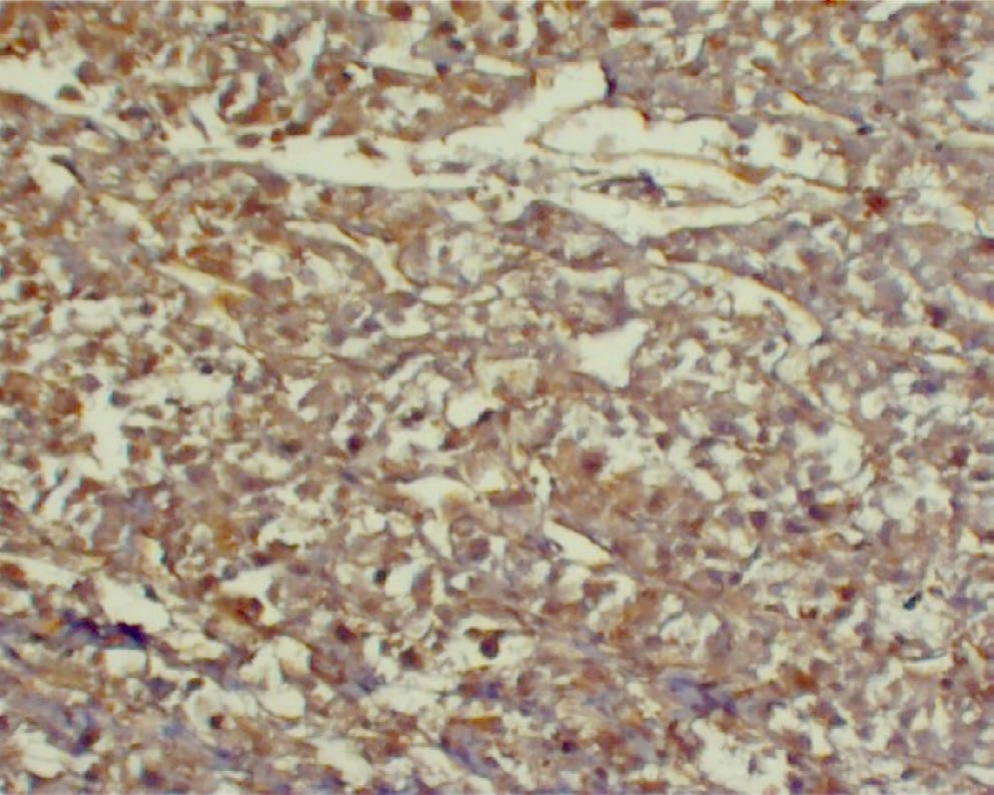
Immunohistochemical study of the adrenal specimen: Positive vimentin stain is noted

The abdominopelvic computerized tomography (CT) scan revealed bilateral manifestations of pheochromocytoma: a hypodense mass with ring enhancement in the right hepatorenal space with fat stranding in pre‐renal fat (size: 113 × 110 mm) and a hypodense mass in the location of the left adrenal gland (size: 142 × 109 mm). Then, the patient underwent laparotomy and tumor resection. The histopathological examination of tissues showed prominent foci of necrosis and focal desmoplastic stroma, which predominantly had foci of a sheet‐like pattern. IHC staining was positive for vimentin, SYN (Figures 2, 3, 4), Ki67 (about 5%‐6% in some areas), S100 (in some tumor cells), and creatine kinase (CK, weakly positive). Also, it was negative for carcinoembryonic antigen (CEA), epithelial membrane antigen (EMA), calretinin, desmin, renal cell carcinoma (RCC), inhibin, LCA, CD3, CD20, CD10, and HMB‐45.

The entirety of pathological examination was in line with our previous diagnosis of malignant pheochromocytoma. According to the Pheochromocytoma of the Adrenal gland Scaled Score (PASS), both masses had a score of 9, indicating a potential for aggressive behavior.

The patient received 17 cycles of systemic cytotoxic chemotherapy with cyclophosphamide, vincristine, and dacarbazine regimen. But unfortunately, painful right globe tumor did not show any significant response to this treatment. Thus, brachytherapy technique was applied to the ocular tumor margin in nine 4 Gy bid fractions over five consecutive days as a palliative measure. One year after this treatment, urine metanephrines and VMA, as well as imaging studies, remained unchanged, no new metastasis was suspected, the patient's ocular pain was improved, and the right eye vision remained unaffected. The final diagnosis of this case was nonfunctioning to barely functioning malignant pheochromocytoma with metastases to the eye muscle.

## 3 DISCUSSION

The management of huge malignant pheochromocytoma is especially challenging because of its extreme rarity and variable clinical course.^3^ Pheochromocytomas are usually suspected based on paroxysmal hypertension, sweating, and palpitation and confirmed by elevated urine metanephrines, dopamine, and VMA levels. In rare cases (as is discussed above), it can manifest without clinical symptoms and be detected initially as a metastatic tumor in an unusual site.^1^, ^2^, ^3^, ^4^ The behavior of the disease is highly variable, and the treatment should be individualized. Unfortunately, because of its rarity, there is not sufficient collective experience in the management of this condition.^3^, ^4^, ^5^, ^6^

The most common metastatic sites of malignant pheochromocytoma are as follows: bone, intra‐abdominal organs, lungs, and pleura.^5^, ^6^, ^7^, ^8^, ^9^, ^10^, ^11^, ^12^, ^13^ Although skull metastasis is quite common,^2^ there has never been a reported case of ocular muscle involvement as the initial presentation of malignant pheochromocytoma. The most similar case was reported by Scharf et al, which was a case of extra‐adrenal pheochromocytoma (in the bifurcation of the abdominal aorta) with right exophthalmos due to orbital metastasis of pheochromocytoma 8 years after mass resection. Interestingly, the patient's blood pressure was significantly increased after right eye massage.^14^

It is nearly impossible to distinguish between malignant and benign pheochromocytoma based on clinical, radiological, or histopathological findings alone. Therefore, the disease is often diagnosed based on multidisciplinary approaches, and the only definitive finding is the presence of metastasis or recurrence in nonchromaffin tissues.^6^, ^7^ In MRI studies, the metastatic lesion of the right eye showed abnormal mass and abnormal enhancement of the right inferior rectus muscle with high attenuation and signal intensity in T2 imaging and contrast‐enhanced T1 imaging, which is the characteristic of adrenal malignancies.^1^, ^8^

Despite the lack of definite criteria for the detection of malignancy, there are some histopathological findings such as nuclear pleomorphism, cellular hyperchromatism, bizarre mitotic figures, and vascular and capsular invasion.^9^, ^10^ Unfortunately, these findings have poor predictive value, because they are also seen in benign lesions. Immunohistochemical staining is not helpful in the prediction of the biological behavior of pheochromocytoma.^11^, ^12^ However, Kumaki et al recently suggested MIB‐1 immunostaining as a useful marker for malignancy prediction.^8^ In their case, the endocrinological abnormalities were minimal, so there was no elevation in blood pressure or heart rate, but due to the size of the mass, the patient reported a dull pain in his abdomen.

Similar to our study, Agarwal et al reported 9 patients from 45 patients (20%) with pheochromocytoma as normotensive pheochromocytomas.^13^ Among those patients, the most common symptom was abdominal pain. It can be noted that almost all of them had increased levels of metanephrine. Some hypotheses were suggested for the mechanism of normotensive pheochromocytomas, including different blood pressure basis and/or variable cardiovascular response to catecholamine and metanephrine released among different individuals. Thus, in the same elevated levels of catecholamines, different blood pressure values were observed.^9^, ^10^, ^11^, ^12^, ^13^ Also, we assume that in huge malignant pheochromocytomas the nature and function of tumor changes and severe malignancy process lead to poor differentiation of chromaffin cells, contributing to the advent of a nonfunctional or minimally functional tumor. Nonetheless, some studies hypothesized that the size of the tumor and its metabolic rate relative to its size are also important factors in the levels of catecholamine products and the clinical picture presented.^6^, ^7^, ^8^, ^9^, ^10^, ^11^, ^12^, ^13^ We believe that this linear relationship does not continue until the late stages, in which because of tumor necrosis and the undifferentiated nature of advanced tumors, the metanephrine levels drop so much that they could be in the upper limits of normal (like our case) or even less.

In our case, many different radiological studies were performed, but the diagnosis was difficult and finally confirmed by pathological examination.

## 4 CONCLUSION

This case was a unique presentation of malignant pheochromocytoma with painful eye movement being the first presentation and classic symptoms such as hypertension being absent. Although there are no definitive criteria for the diagnosis and management of malignant pheochromocytoma, it seems that serial radiological examinations with close follow‐up and pathological studies are suitable for the diagnosis and management of difficult clinical situations.

## CONFLICT OF INTEREST

There are no conflicts of interest.

## AUTHOR CONTRIBUTIONS

GK, AT, AB, PN, AMP, NJ, and RB: contributed to the initial diagnostics and treatment of the patient. All authors wrote and edited the manuscript.
